# Decreased Body Mass Index in Schoolchildren After Yearlong Information Sessions With Parents Reinforced With Web and Mobile Phone Resources: Community Trial

**DOI:** 10.2196/jmir.5584

**Published:** 2016-06-24

**Authors:** Jenny Vilchis-Gil, Miguel Klünder-Klünder, Ximena Duque, Samuel Flores-Huerta

**Affiliations:** ^1^ Hospital Infantil de México Federico Gómez, Ministry of Health (SSA) Community Health Research Department Mexico City Mexico; ^2^ Mexican Institute of Social Security Unit of Medical Research in Infectious and Parasitic Diseases Mexico City Mexico

**Keywords:** obesity, child, early intervention (education), prevention, Internet, mobile phone

## Abstract

**Background:**

The obesity pandemic has now reached children, and households should change their lifestyles to prevent it.

**Objective:**

The objective was to assess the effect of a comprehensive intervention on body mass index *z*-score (BMIZ) in schoolchildren.

**Methods:**

A yearlong study was conducted at 4 elementary schools in Mexico City. Intervention group (IG) and control group (CG) were split equally between governmental and private schools. Three educational in-person parents and children sessions were held at 2-month intervals to promote healthy eating habits and exercise. To reinforce the information, a website provided extensive discussion on a new topic every 2 weeks, including school snack menus and tools to calculate body mass index in children and adults. Text messages were sent to parents’ mobile phones reinforcing the information provided. The IG contained 226 children and CG 181 children. We measured their weight and height and calculated BMIZ at 0, 6, and 12 months.

**Results:**

The CG children showed a change of +0.06 (95% CI 0.01, 0.11) and +0.05 (95% CI 0.01, 0.10) in their BMIZ at 6 and 12 months, respectively. The BMIZ of IG children decreased by -0.13 (95% CI -0.19 to -0.06) and -0.10 (95% CI -0.16 to -0.03), respectively, and the effect was greater in children with obesity.

**Conclusions:**

The comprehensive intervention tested had beneficial effects, preserved the BMIZ of normal weight children, and reduced the BMIZ of children with obesity.

## Introduction

Childhood obesity is a public health problem because it is associated with comorbidities such as metabolic syndrome, hypertriglyceridemia, and type 2 diabetes [1-3]. Therefore, it is important to develop alternative strategies to prevent this health problem from an early age. In the last two decades, overweight and obesity have increased in Mexican children aged 5-11 years. The national nutrition surveys showed that from 1999 to 2006 the prevalence increased from 26.9% to 34.8%. In 2012, the prevalence was 34.4% [4], indicating that this problem prevails with unacceptably high figures for this age group [5].

Metabolically, obesity is caused by an imbalance in which energy intake exceeds expenditure in a persistent fashion. Low energy expenditure is caused by decreased physical activity and increased sedentary habits [6-8]. To neutralize this problem and its comorbidities, intervention studies have been performed to change lifestyles of eating and physical activity, but many have been conducted in minority populations [9] without a control group [10,11] for short periods [9,12] and have had no lasting effect on the body mass index *z*-scores (BMIZ) [13,14]. The energy imbalance of overweight children results from the unhealthy lifestyles acquired at home, and health providers need to inform parents on how to change unhealthy feeding habits and sedentary habits for better alternatives.

Studies in children and adolescents have shown that Web-based information improves people’s knowledge, attitudes, and behaviors related to feeding and physical activity [15,16]. However, such information is rarely a comprehensive educational intervention [10] that also incorporates sociocultural and economic conditions of the target group. Furthermore, there is a need to educate parents about their role in the prevention of obesity. It is expected that information would change feeding behaviors, the types of foods available at home, and preparation and enjoyment of healthy menus. Currently, the Internet and mobile phones are regular informational tools of many households in Mexico City with several apps available for users. In Mexico, the percentage of Internet users nationwide grew from 19.5% to 40.2% from 2006 to 2012, the largest number being in urban areas. In the same period, the rate of mobile phone users increased from 52.6% to 90.8% [17]. One advantage of mobile phones for educational purposes is the instantaneous relay of short messages at any time and place.

The aim of this study was to compare the daily nutrition-based activities performed in schools in Mexico City, such as the availability of healthy food and drinking water [18,19], with an educational intervention involving in-person sessions with parents, distance activities using Web resources and mobile phone reminders, and printed materials in order to improve or at least preserve the BMIZ of Mexican children in elementary schools.

## Methods

### Design and Study Population

The study was approved by Research, Ethics, and Biosafety Committees of the Federico Gomez Children’s Hospital of Mexico (HIMFG in Spanish), a National Institute of Health. After the Ministry of Public Education granted the authorization to perform the study, 4 elementary schools from a middle-class suburb of Mexico City with similar number of students were included in the study. All school principals granted authorization for the study. The intervention was implemented in 2 schools, 1 governmental and 1 private (intervention group, IG), whereas the other 2 schools, 1 governmental and 1 private, were controls (control group, CG). Before implementation, educational materials and the website were designed and developed, which were the tools of the intervention. The subsequent activities are described as phase 1 of the study. The grades that participated were the first to fourth. Boys and girls were included, regardless of their BMIZ. Before starting the study, both objectives and activities were explained to teachers, parents, and children and also verbal agreement and written informed consent was obtained from children and their parents, respectively.

### Phase 1: Design and Development of Educational Materials and Website

#### Artwork That Would Accompany the Messages

The artworks for this project, such as images of children eating healthy foods, were created by the designers of Universum, the Science Museum of the National Autonomous University of Mexico (UNAM). Designs were considered to be ad hoc for the age of the children and culture of Mexico City.

#### Informative Topics, Posters, and Messages

Twenty topics were developed to inform parents about overweight, obesity, healthy eating, physical activity, and health risks that obesity involves, which are described in Table 1 [20-25]. The topics were developed by pediatricians, nutritionists, nurses, and physical educators, who were asked to adjust the information to the following format: not more than 500 words, to suggest activities that could be implemented by the family, and annotate 2 or more hyperlinks to obtain more information, if desired. Topics were edited by a pediatric nutrition expert, for the purpose of standardizing the language and isolating 2 brief messages that would be sent to the parents' mobile phones. Information posters were developed to reinforce the written topics, which were placed in strategic locations within the intervention schools.

Various materials were developed for the children to take home, such as laminated place mats with the images of the Mexican Eatwell Plate [26] and Drinkwell Pitcher [27] and the pyramid of physical activity. For illustrative purposes, some screenshots of the educational materials used in the intervention are presented in Multimedia Appendix 1 (Selected screenshots of the materials used in the intervention); additionally, the authors can be contacted to obtain samples of the materials.

Guidelines for parents were developed with information on how to prepare a healthy school lunch, including numerous examples.

**Figure 1:**
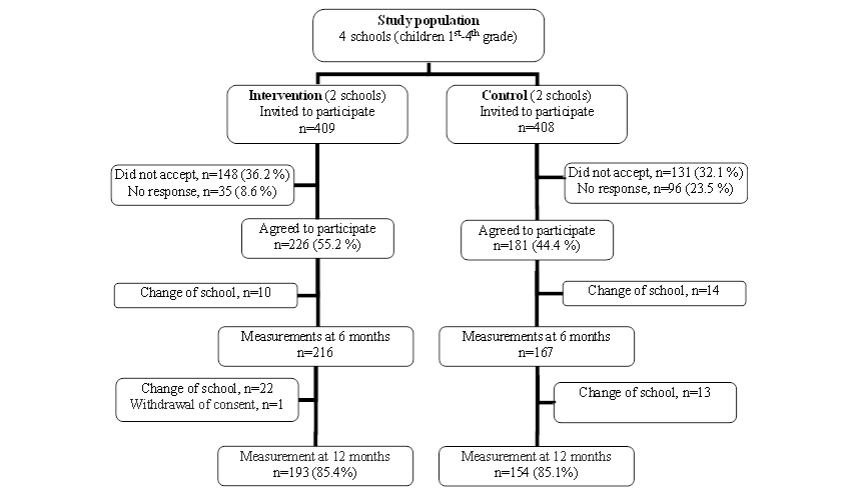
Study population: screening results and follow-up.

**Table 1:** Work topics on the website, phone messages, and posters.

Website topics	Messages	Poster
1. What is obesity? Why are there more obese people now than before?	M1, M2	The change in lifestyle has affected our health
2. Obesity and its relationship to chronic degenerative diseases	M3, M4	
3. Lifestyles, feeding, activity/sedentary, and health monitoring with regular measurements	M5, M6	Consequences of overweight and obesity in health
4. Lifestyle and health. We stay as we are or we make an effort to improve the health of the family and of each of its members	M7, M8	
5. Eating breakfast and bringing lunch to school are essential for health and school performance of children	M9, M10	Breakfast at home and lunch at school
6. Physical activity is much more than expending energy	M11, M12	
7. Sedentary lifestyle, a risk for health	M13, M14	Free and spontaneous play
8. Measure yourself. Self-measurement is much more than knowing your weight	M15, M16	
9. Plain water is the healthiest	M17, M18	How can we avoid a sedentary lifestyle?
10. Not all the energy that we consume is the same in terms of health	M19, M20	
11. Carbohydrates. Health benefits and risks	M21, M22	Natural water is the healthiest drink
12. Fiber. Health benefits and risks	M23, M24	
13. Importance of eating fruits and vegetables	M25, M26	Fruits and vegetables in my feeding
14. Lipids. Health benefits and risks	M27, M28	
15. Proteins in food. Health benefits and risks	M29, M30	Varied feeding
16. Table salt in food. Health benefits and risks	M31, M32	
17. Vitamins and minerals. Health benefits and risks	M33, M34	Natural foods better than processed foods
18. Family behavior during food consumption at home	M35, M36	
19. Planning food purchases. Learning to read labels on processed and industrialized foods	M37, M38	
20. Integration. Create a healthy eating environment with your family	M39, M40	

#### Design and Development of the Website

The project website was nested in the official HIMFG website www.himfg.edu.mx. The picture of 2 children eating healthy foods was the identification icon of the project. Upon accessing the site, the user found the biweekly highlighted topic and the following icons:

1. Previous topics. By accessing this icon, the list of biweekly topics appeared, which could be accessed at any time. At the end of 2 weeks, the current theme was stored in this folder.

2. School snacks. This icon contained information on how to develop a healthy school snack and 25 examples of school snacks.

3. Guidelines on healthy eating and physical activity. This icon contained files of 2 guides on nutrition and physical activity; these printed guides were also sent to parents through their children.

4. Posters. This folder contained the posters hung at schools on a monthly basis. Each poster reinforced the current theme. A total of 9 posters were placed.

5. Software for the calculation and interpretation of body mass index (BMI). This icon invited parents to assess the BMI of themselves and their children. When they entered a weight and height, the program returned a BMI, indicating whether there was a health risk.

6. Contact, questions, and comments. In this section, parents could contact researchers to raise questions or make comments related to the project.

7. Window for researchers. The page included a window that allowed researchers to activate the user, evaluate the number of accesses to the site by each of the families, read the comments or questions that were raised by parents, and keep the page updated with the information of each of the icons. A visual of the website is shown in Multimedia Appendix 2 (Selected screenshot of the website).

### Phase 2: Intervention Implementation

The intervention was implemented from October 2013 to July 2014; parents and all children, regardless of their BMIZ, participated in the intervention.

#### Activities of Parents in the Intervention Group

**1. Website**. An interactive workshop to learn and use the project website was developed. The name of their child was the username, and the password was the date of birth of their child. Parents were invited to access the site at least once every 2 weeks and review the current topic.

** 2. Messages by mobile phone.** A short message (25 words on average) was sent to the parents' mobile phone, every week. The message was related to the current topic of the website that motivated and reinforced behavior changes. A total of 40 messages were sent (Multimedia Appendix 3: Selected screenshots of examples of messages sent to parents' mobile phone).

**3. In-person activities.** Parents from the intervention schools were invited to 3 sessions of 1 hour each, once every 2 months, for the purpose of giving them feedback on topics of eating and physical activity that were on the website, to participate in some project activities, and to answer their questions and receive comments to modify or improve the website.

#### Activities of Children in the Intervention Group

**1. Workshops.** Two nutritionists and a physical educator conducted 4 workshops, 1 every 2 months, with a duration of 1.5 hours. The workshops were integrated with both board games and physical games and with educational materials to reinforce healthy eating habits and physical activity.

**2. Educational materials.** They were provided with board games and plastic place mats with pictures of a healthy dish [26], the pyramid of physical activity, and a picture of a healthy drink pitcher [27].

**3. Visit to Universum Museum.** Children and parents visited the Life in Balance room of Universum, the Science Museum of the UNAM.

**4. Posters.** Each month, in a visible area and an area with large influx of children inside the school (eg, at the entrance of the school and in the schoolyard) a new poster alluding to the project and specifically the current website topic was placed.

### Measurements

#### Sociodemographic Information

Information about the socioeconomic status was obtained by administering a questionnaire to know the number of children, parental education, and housing characteristics.

#### Anthropometric Measurements

Weight, height, and waist circumference were measured in schoolchildren of all schools at baseline and then at 6 and 12 months. Weight was measured with a digital scale (Seca 882; Seca Corp, Hamburg, Germany) with an accuracy of 0.1 kg. Height was measured with a stadiometer (Seca 225; Seca Corp, Hamburg, Germany) with an accuracy of 0.1 cm. Waist circumference was measured with a nonelastic flexible measuring tape (Seca 200). Two trained nutritionists took these measurements according to international procedures [28]. Briefly, the children were measured without shoes and in light clothing, standing in the center of the scale platform or stadiometer, arms resting loosely on the sides, and head positioned in the Frankfort plane. To measure waist circumference, the child climbed an anthropometric box 60 cm tall, the tape was placed at the midpoint between the iliac crest and the lower costal margin, and the reading was taken at the end of a normal exhalation. The purpose of having the child stand on the box was to bring the reader's eyes close to the height of the tape to avoid parallax errors.

Body mass index (BMI) and BMI *z*-score (BMIZ) are measures used to define obesity. BMI is a measure of weight adjusted for height. It is calculated as weight in kilograms divided by the square of height in meters. Whereas in adults the BMI cut points that define obesity and overweight are not linked to age and do not differ for males and females, in growing children BMI varies with age and sex. For BMI to be meaningful in children it must be compared to a reference-standard that accounts for child age and sex. Body mass index *z*-scores, also called BMI standard deviation (SD) scores, are measures of relative weight adjusted for child age and sex. Given a child's age, sex, BMI, and an appropriate reference standard, a BMI *z*-score (or its equivalent BMI-for-age percentile) can be determined.

 Children were classified according to the nutritional status as underweight (*z*-score ≤ -2), normal weight (-2< *z*-score <1), overweight (1≤ *z*-score <2), or obese (*z*-score ≥2), using as a reference the data from the World Health Organization report of 2007 [29]. Additionally, the waist circumference percentile was calculated with consideration of age, sex, and height, using the tables for Mexican children [30].

After each of the anthropometric measurements, both groups of children were handed a letter with the results of the nutritional status and tips to maintain or improve their health. The recommendations mainly focused on eating and physical activity habits.

### Data Analyses

Descriptive statistics were used to describe the baseline of the study population. The mean weight and height were adjusted for age and sex by multiple linear regression. The socioeconomic variable was built with the information obtained from the questionnaire on housing characteristics and ownership of property. Households were grouped into tertiles according to their score for socioeconomic status. To compare the groups at baseline, *t* test for independent data was used for continuous variables and the χ^2^ test was used for categorical data.

Because, frequently in this type of study, participants do not always follow instructions and consequently adherence is never 100%, we considered it appropriate to perform the data analysis by intention-to-treat, in which all participants assigned to each group are analyzed regardless of their adherence.

The intragroup BMIZ changes from baseline to 6 months and from baseline to 12 months were compared using paired *t* test. To compare the intergroup BMIZ change, *t* test for independent data was used.

A model of mixed-effects linear regression was used to assess the change in BMIZ during follow-up. Because children are nested within schools, a mixed model with 3 levels and random intercepts by subject and school were tested. The model was adjusted by the fixed variables of age and BMIZ at baseline. The interaction between the intervention group and the time at each evaluation, 6 and 12 months, was assessed. Mean BMIZ by group (intervention or control) and by time was calculated and a graphic was done using marginal analysis. *P* values < .05 were considered statistically significant for all analyses. The analysis was performed using Stata v12.0 (StataCorp, College Station, TX, USA).

## Results

The number of schoolchildren who participated was 226 in the IG and 181 in the CG. The participation rates were 55.2% (226/409) in IG and 44.4% (181/408) in CG. At 12 months, 85% of children in both groups completed the measurements. The proportion of children who did not conclude the study can be attributed to children moving to different schools for personal reasons. However, there was no movement of children between study groups (Figure 1).

The development of the intervention is presented in Table 2; among the parents, 51.3% (116/226) attended at least one educational session, 40.7% (92/226) consulted the website, and 91.2% (206/226) received mobile phone messages. All children handed in materials and participated in workshops.

**Table 2:** Intervention development during the school year (n=226).

Intervention			n (%)
**Parents' sessions**				
	None		110 (48.7)
	At least one session		116 (51.3)
		1 session	78 (34.5)
		2 sessions	34 (15.0)
		3 sessions	4 (1.8)
**Consultation of the website**				
	No		134 (59.3)
	Yes		92 (40.7)
**Sending messages to mobile phone**				
	No message		20 (8.8)
	Message		206 (91.2)

Table 3 presents the baseline characteristics of the study population. The demographic, anthropometric, and socioeconomic characteristics of both groups were similar. The average age was 8 years, and there was no difference in BMIZ. The only exception was the use of mobile phones, which was significantly higher in the IG.

**Table 3:** Baseline characteristics of the study population according to study group.

Characteristics					Control n=181		Intervention n=226	*P* ^a^		
**Age in years, mean (SD)**					8.1 (1.2)		7.9 (1.2)	.13
**Sex, female, n (%)**					90 (49.7)		101 (44.7)	.31
**Anthropometric measurements**									
		Weight^b^, kg, mean (SD)			30.1 (5.1)		29.3 (5.0)	.13
		Height^b^, cm, mean (SD)			127.4 (7.5)		126.4 (7.4)	.14
		BMI^c^*z*-score^d^, mean (SD)			0.98 (1.3)		0.85 (1.4)	.35
		BMI classification^d^, n (%)						
			Normal (-2≤ *z*-score <1)		89 (49.2)		129 (57.1)			
			Overweight (1≤ *z*-score <2)		46 (25.4)		52 (23.0)	
			Obese (*z*-score ≥2)		45 (25.4)		45 (19.9)	.25
		Waist circumference, percentile, mean (SD)			53.0 (21.8)		52.2 (21.2)	.69
**Maternal schooling, n (%)**									
		Secondary or less			24 (14.1)		42 (20.8)		
		High school or technical school			77 (45.3)		76 (37.6)	
		College career or postgraduate			69 (40.6)		84 (41.6)	.16
**Number of children, n (%)**									
		1-2			116 (72.1)		153 (76.1)	
		≥3			45 (27.9)		48 (23.9)	.38
**Socioeconomic status (tertiles), n (%)**		
		Lower			42 (26.1)		63 (32.1)
		Medium			53 (32.9)		76 (38.8)	
		Higher			66 (41.0)		57 (29.1)	.06
**Availability of Internet, n (%)**									
		At home			129 (86.6)		141 (82.9)	.37
		On the mobile phone			94 (63.0)		92 (54.1)	.11
**With mobile phone, n (%)**									
		Mothers			146 (85.4)		190 (92.2)	.03
		Parents			155 (90.1)		201 (95.3)	.05
			

^a^*t* test and χ^2^ test.

^b^ Means adjusted by age and sex.

^c^ BMI: body mass index.

^d^ World Health Organization, 2007.

Table 4 shows the differences in BMI and BMIZ both within and between groups from baseline to 6 months and from baseline to 12 months. Intragroup analysis showed that in the IG, BMIZ decreased from 0 to 6 months (-0.07, 95% CI -0.12 to -0.03) and was maintained at 12 months. Whereas BMIZ of the CG increased (0.07, 95% CI 0.02, 0.12) from baseline to 6 months and persisted until 12 months. When stratified by nutritional status according to baseline BMIZ, the BMIZ of CG children with normal weight increased at 6 months (*P*=.002) and was maintained at 12 months (*P*=.04), whereas the BMIZ of the IG was maintained throughout the follow-up. Overweight children of both groups did not show significant changes from baseline to 6 months or from baseline to 12 months. Concerning obese children, those of CG maintained their BMIZ throughout the follow-up, whereas BMIZ of IG children decreased at 6 months (*P*<.001) and continued decreasing to 12 months (*P*=.001).

When comparing the BMIZ change between IG and CG in children who started the study with normal weight, differences observed between groups at 6 and 12 months were -0.17 (95% CI -0.27 to -0.07) and -0.12 (95% CI -0.24 to -0.01), respectively. In children who were overweight at baseline, an effect between groups of -0.15 (95% CI -0.24 to -0.01) at 6 months was observed, although this effect was not maintained at 12 months. In obese children, the effect on BMIZ between groups was -0.12 (95% CI -0.23 to -0.02) at 6 months and -0.16 (95% CI -0.32 to -0.01) at 12 months.

**Table 4:** Intra- and intergroup body mass index *z*-score comparison during follow-up (intervention group, n=191; control group, n=154).

Characteristics			0 months	6 months	12 months	∆ 0 to 6 months	*P* ^a^	∆ 0 to 12 months	*P* ^a^
			Mean (SD)	Mean (SD)	Mean (SD)	Mean (95% CI)		Mean (95% CI)	
**BMI^b^ (kg/m^2^)**								
	Control		18.4 (3.2)	18.8 (3.3)	19.1 (3.5)	0.50 (0.38 to 0.60)	<.001	0.77 (0.62 to 0.92)	<.001
	Intervention		17.9 (3.3)	18.0 (3.4)	18.4 (3.6)	0.13 (0.03 to 0.23)	.01	0.50 (0.36 to 0.63)	<.001	
**BMIZ^c,d^**								
	Control		1.01 (1.3)	1.08 (1.3)	1.06 (1.3)	0.07 (0.02 to 0.12)	.004	0.05 (-0.01 to 0.11)	.07
	Intervention		0.85 (1.3)	0.77 (1.3)	0.80 (1.3)	-0.07 (-0.12 to -0.03)	.002	-0.05 (-0.10 to 0.01)	.08
**BMIZ according to baseline nutritional status^d^**								
	Normal weight								
		Control	-0.15 (0.7)	-0.01 (0.8)	-0.05 (0.8)	0.13 (0.05 to 0.21)	.002	0.10 (0.01 to 0.19)	.04
		Intervention	-0.13 (0.7)	-0.17 (0.7)	-0.16 (0.8)	-0.04 (-0.10 to 0.02)	.20	-0.02 (-0.10 to 0.05)	.46
		Difference IG^e^ versus CG^f^				-0.17 (-0.27 to -0.07)	<.001	-0.12 (-0.24 to -0.01)	.03
	Overweight								
		Control	1.43 (0.3)	1.52 (0.4)	1.52 (0.5)	0.09 (-0.01 to 0.18)	.07	0.09 (-0.03 to 0.21)	.15
		Intervention	1.52 (0.3)	1.46 (0.4)	1.56 (0.4)	-0.06 (-0.16 to 0.03)	.20	0.05 (-0.06 to 0.15)	.36
		Difference IG versus CG				-0.15 (-0.24 to -0.01)	.03	-0.04 (-0.20 to 0.11)	.59
	Obesity								
		Control	2.64 (0.5)	2.60 (0.5)	2.58 (0.5)	-0.05 (-0.11 to 0.02)	.14	-0.06 (-0.15 to 0.04)	.22
		Intervention	2.78 (0.6)	2.61 (0.7)	2.55 (0.7)	-0.17 (-0.26 to -0.08)	<.001	-0.22 (-0.35 to -0.09)	.001
		Difference IG versus CG				-0.12 (-0.23 to -0.02)	.02	-0.16 (-0.32 to -0.01)	.04

^a^ Paired *t* test and *t* test for independent samples.

^b^ BMI: body mass index.

^c^ BMIZ: body mass index *z*-score.

^d^ World Health Organization, 2007.

^e^ IG: intervention group.

^f^ CG: control group.

**Figure 2:**
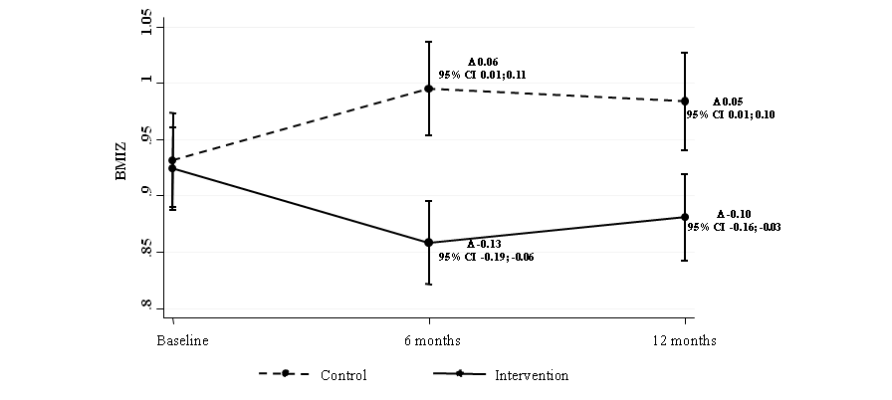
Body mass index *z*-score (BMIZ) change in children during follow-up. Linear regression mixed effects model with random intercept, adjusted for baseline age and baseline BMIZ.

On the basis of a linear regression model with random intercept mixed effect and adjusted by baseline age and BMIZ, the graph shown in Figure 2 was constructed. The continuous line in this figure shows that in the first 6 months the intervention had a greater effect on the BMIZ of schoolchildren; whereas BMIZ increased in the CG children (dashed line). At baseline, IG and CG children began with a similar BMIZ; however, the mean of BMIZ changed at 6 and 12 months between groups. The adjusted means of BMIZ of the CG were estimated at 0.93 (95% CI 0.89-0.97), 0.99 (95% CI 0.95-1.04), and 0.98 (95% CI 0.94-1.03) at baseline, 6 months, and 12 months, respectively; those of IG were estimated at 0.92 (95% CI 0.89-0.96), 0.86 (95% CI 0.82-0.89), and 0.88 (95% CI 0.84-0.92), respectively.

## Discussion

This study shows the results of an intervention implemented in elementary schools comprising multiple components. The intervention included in-person and remote activities aimed at parents and children. Comparing the BMIZ during the study with the BMIZ at baseline, in the intervention group BMIZ decreased and in the control group, BMIZ increased. In addition, the intervention maintains the BMIZ of normal weight children and decreased the BMIZ of obese children. Moreover, it is pertinent to emphasize that the strategy described in this report was not intended to treat obesity but sought to reach as many parents as possible to promote changes in the eating and physical activity habits of their children regardless of BMIZ.

For the management of obese adolescents and adults, the effectiveness of sending information electronically versus individual face-to-face consultation has been compared, and weight reduction is greater in those receiving individual consultation [31]. However, such consultations become unsustainable because of the magnitude of the problem and also because each subject requires a large amount of attention and resources. In this study, the effect of the yearlong follow-up intervention in the whole sample was a BMIZ reduction of 0.10, equivalent to a decrease of 0.4 kg. It is important to mention that studies with similar interventions achieved beneficial effects on BMIZ, whereas others achieved positive changes only in eating and physical activity habits and not the BMIZ [17,32,33].

In this report, when the effect was analyzed by subgroups considering the initial BMIZ, the intervention had a differential effect: the BMIZ of IG children with normal weight was maintained during the follow-up, whereas BMIZ of children from the CG increased. The obese children from the IG reduced 0.22 of BMIZ by 12 months; this effect was greater than that observed in obese children of the CG (-0.06) and the whole population of the intervention group (-0.10). These results are similar to those of a Chilean intervention study that found that obese schoolchildren are the subpopulation with better responses [34].

In our study, in the children with overweight in the intervention group the BMIZ decreased at 6 months (intervention effect) but this effect disappeared at 12 months; this may be because the parents of these children do not yet perceive the health problem in which their children are immersed, and returned to their usual habits.

In the IG, the greatest reduction of BMIZ was observed in the first 6 months but did not continue to decrease at 12 months. However, in the subgroup of obese children the BMIZ continued improving after 6 months. This effect has been reported in other study that used a website to send information to parents [35]. The question remains as to how much longer the effect of the intervention can last, especially in obese children who continue to improve their BMIZ.

Concerning obese children of the CG who maintained their BMIZ (rather than increasing it), we propose that parents could have changed to healthier habits after receiving a letter with interpretation of their children’s anthropometric measurements and health care recommendations. It has been reported that regular anthropometric measures can improve or reduce BMI [36].

Regardless of the specific intervention, it seems that a crucial element of our strategy was that it targeted parents, who play a central role in promoting healthy habits within the family [33]. Similar results have been reported by Haerens et al [33] in a 2-year follow-up study using electronic means (but no website information) who found that parental involvement was a key factor to maintain BMIZ values. In other studies, in which information was sent to parents via a website, a direct association between the number of visits to the website and reduction in BMIZ was observed [15,16,37]. In this context, parents who most often consult the website are presumably the most informed and implement more changes in their children’s eating habits and physical activity.

In our study, the parents of 91.2% (206/226) of IG children received text messages, the highest coverage of all the activities of the strategy. Each of the phone messages summarizes a topic that was extensively explained on the website. The use of mobile phones could play an important role in making successful interventions because other studies have found stronger communication effects using text messages versus the Internet [38]. Additionally, the use of mobile phones has achieved great efficacy at promoting improvements in maternal and child health in resource-poor countries such as India [39], and perhaps this device could be useful in crowded cities with scarce means of transportation.

The main limitations arise in our study: (1) The assignment of participating schools was not randomized. Despite this problem, the BMIZ of the schoolchildren of the IG and CG were comparable at baseline and the effect of the intervention was assessed with the BMIZ change (Δ) during the course of the study. (2) The study participation was approximately 50% and the reasons for nonparticipation should be explored in future studies. Nevertheless, parents of the 2 groups who agreed to participate remained in the study in relatively high proportion at 12 months. (3) The changes in dietary intake and physical activity of participants are not included in this report, and we can only assume that the changes in BMIZ of children were due to the improvement of these habits. (4) The effect of each of the components on BMIZ modification was not evaluated separately in this study because neither in-person activities nor the website consultation showed 100% parental participation. However, the positive effect observed in both normal weight children and obese children of IG possibly is due to the intervention.

The following are the strengths of this study: (1) The study sent information to parents by several channels, in person, through children, via the Internet, and via mobile phones. The few in-person sessions allowed parents a more efficient use of their time in a large, complex city in which in-person meetings are difficult. (2) The follow-up lasted 12 months, longer than in other studies in which interventions last only a few months. (3) Although the intervention had multiple components, it was affordable. (4) The intervention was not only addressed to the obese population but also involved schoolchildren of all BMI classes. This allowed us to determine that, although the effect of the intervention was greater in children with obesity, children with normal weight maintained their BMI, indicating that exposure to obesity risk factors could be decreased with this type of intervention.

Fighting overweight and obesity is a complex undertaking, and some intervention studies in schools have shown effectiveness in restraining but not reducing obesity [34]. Other studies have achieved behavioral changes without reducing BMIZ, and further studies focused on prevention rather than obesity treatment are currently required.

Although the results of this study cannot be generalized, the intervention is promising for implementation of strategies at a distance. Sending information via the Web and smarter mobile phones could encourage better eating and physical activity habits with the aim of preserving or improving children’s BMI. In large cities where mobility is difficult but the use of the aforementioned devices has increased [11], it could be useful to take advantage of this kind of intervention for the well-being of schoolchildren.

Lastly, serious and accessible information to promote healthy habits at home, which comes from the school itself, supported by a health institution such as HIMFG, always will be a counterbalance to the commercial information given by the media.

The comprehensive intervention combining in-person activities with Web-based information and mobile phone messaging maintained the BMIZ of normal weight children and decreased the BMIZ of children with obesity. Thus, this can be an affordable alternative to promote health changes in children at the household level.

## Appendix

Multimedia Appendix 1 Selected screenshots of the materials used in the intervention.

Multimedia Appendix 2 Selected screenshot of the website.

Multimedia Appendix 3 Selected screenshots of examples of messages sent to parents' mobile phone.

## Abbreviations

BMI: body mass index
BMIZ: body mass index *z*-score
CG: control group
IG: intervention group
HIMFG: Federico Gomez Children’s Hospital of Mexico
UNAM: National Autonomous University of Mexico

## References

[1] Freedman DS, Khan LK, Dietz WH, Srinivasan SR, Berenson GS (2001). Relationship of childhood obesity to coronary heart disease risk factors in adulthood: the Bogalusa Heart Study. Pediatrics.

[2] Owen CG, Whincup PH, Orfei L, Chou Q, Rudnicka AR, Wathern AK, Kaye SJ, Eriksson JG, Osmond C, Cook DG (2009). Is body mass index before middle age related to coronary heart disease risk in later life? Evidence from observational studies. Int J Obes (Lond).

[3] Messiah SE, Arheart KL, Luke B, Lipshultz SE, Miller TL (2008). Relationship between body mass index and metabolic syndrome risk factors among US 8- to 14-year-olds, 1999 to 2002. J Pediatr.

[4] Gutierrez Jp, Rivera-Dommarco J, Shamah-Levy T, Villalpando-Hernandez S, Franco A, Cuevas-Nasu L, Romero-Martinez M, Herandez-Avila M (2012). Encuesta Nacional de Salud y Nutrición 2012. Resultados Nacionales.

[5] Lobstein T, Jackson-Leach R, Moodie ML, Hall KD, Gortmaker SL, Swinburn BA, James WP, Wang Y, McPherson K (2015). Child and adolescent obesity: part of a bigger picture. Lancet.

[6] Martinez JA (2000). Body-weight regulation: causes of obesity. Proc Nutr Soc.

[7] Flores-Huerta S, Klünder-Klünder M, Medina-Bravo P (2008). Elementary school facilities as an opportunity to prevent overweight and obesity in childhood. Bol Med Hosp Infant Mex.

[8] Prentice AM, Jebb SA (1995). Obesity in Britain: gluttony or sloth?. BMJ.

[9] Neville LM, O'Hara B, Milat AJ (2009). Computer-tailored dietary behaviour change interventions: a systematic review. Health Educ Res.

[10] Nguyen B, Kornman KP, Baur LA (2011). A review of electronic interventions for prevention and treatment of overweight and obesity in young people. Obes Rev.

[11] Manzoni GM, Pagnini F, Corti S, Molinari E, Castelnuovo G (2011). Internet-based behavioral interventions for obesity: an updated systematic review. Clin Pract Epidemiol Ment Health.

[12] Waters E, de SA, Hall BJ, Brown T, Campbell KJ, Gao Y, Armstrong R, Prosser L, Summerbell CD (2011). Interventions for preventing obesity in children. Cochrane Database Syst Rev.

[13] Monasta L, Batty GD, Macaluso A, Ronfani L, Lutje V, Bavcar A, van Lenthe FJ, Brug J, Cattaneo A (2011). Interventions for the prevention of overweight and obesity in preschool children: a systematic review of randomized controlled trials. Obes Rev.

[14] Brown T, Summerbell C (2009). Systematic review of school-based interventions that focus on changing dietary intake and physical activity levels to prevent childhood obesity: an update to the obesity guidance produced by the National Institute for Health and Clinical Excellence. Obes Rev.

[15] Delamater AM, Pulgaron ER, Rarback S, Hernandez J, Carrillo A, Christiansen S, Severson HH (2013). Web-based family intervention for overweight children: a pilot study. Child Obes.

[16] White MA, Martin PD, Newton RL, Walden HM, York-Crowe EE, Gordon ST, Ryan DH, Williamson DA (2004). Mediators of weight loss in a family-based intervention presented over the internet. Obes Res.

[17] Comisión Federal de Telecomunicaciones (COFETEL) (2012). Scribd.

[18] Secretaría de Salud (2010). SEP.

[19] Flores-Huerta S, Klünder-Klünder M, Medina-Bravo P (2011). General guidelines established for the sale or distribution of food and beverages consumed in basic education establishments: critical analysis of the AGREEMENT. Bol Med Hosp Infant Mex.

[20] Reedy J, Krebs-Smith SM (2010). Dietary sources of energy, solid fats, and added sugars among children and adolescents in the United States. J Am Diet Assoc.

[21] Vereecken CA, Todd J, Roberts C, Mulvihill C, Maes L (2006). Television viewing behaviour and associations with food habits in different countries. Public Health Nutr.

[22] Hendrie GA, Coveney J, Cox DN (2012). Defining the complexity of childhood obesity and related behaviours within the family environment using structural equation modelling. Public Health Nutr.

[23] Linabery AM, Nahhas RW, Johnson W, Choh AC, Towne B, Odegaard AO, Czerwinski SA, Demerath EW (2013). Stronger influence of maternal than paternal obesity on infant and early childhood body mass index: the Fels Longitudinal Study. Pediatr Obes.

[24] Melendez G (2008). Factores asociados con sobrepeso y obesidad en el ambiente escolar.

[25] Gidding SS, Lichtenstein AH, Faith MS, Karpyn A, Mennella JA, Popkin B, Rowe J, van Horn L, Whitsel L (2009). Implementing American Heart Association pediatric and adult nutrition guidelines: a scientific statement from the American Heart Association Nutrition Committee of the Council on Nutrition, Physical Activity and Metabolism, Council on Cardiovascular Disease in the Young, Council on Arteriosclerosis, Thrombosis and Vascular Biology, Council on Cardiovascular Nursing, Council on Epidemiology and Prevention, and Council for High Blood Pressure Research. Circulation.

[26] Secretaria de Salud (2013). DOF.

[27] Rivera JA, Muñoz-Hernández O, Rosas-Peralta M, Aguilar-Salinas CA, Popkin BM, Willett WC, Comité de Expertos para las Recomendaciones (2008). Beverage consumption for a healthy life: recommendations for the Mexican population. Salud Publica Mex.

[28] World Health Organization (1995). Physical status: the use and interpretation of anthropometry.

[29] (2007). World Health Organization.

[30] Klünder-Klünder M, Flores-Huerta S (2011). Waist circumference values according to height percentiles: a proposal to evaluate abdominal obesity in Mexican children and adolescents between 6 and 16 years of age. Arch Med Res.

[31] Maggio AB, Saunders GC, Gal-Duding C, Beghetti M, Martin XE, Farpour-Lambert NJ, Chamay-Weber C (2013). BMI changes in children and adolescents attending a specialized childhood obesity center: a cohort study. BMC Pediatr.

[32] Doyle AC, Goldschmidt A, Huang C, Winzelberg AJ, Taylor CB, Wilfley DE (2008). Reduction of overweight and eating disorder symptoms via the Internet in adolescents: a randomized controlled trial. J Adolesc Health.

[33] Haerens L, Deforche B, Maes L, Stevens V, Cardon G, De Bourdeaudhuij I (2006). Body mass effects of a physical activity and healthy food intervention in middle schools. Obesity (Silver Spring).

[34] Kain J, Concha F, Moreno L, Leyton B (2014). School-based obesity prevention intervention in Chilean children: effective in controlling, but not reducing obesity. J Obes.

[35] Williamson DA, Walden HM, White MA, York-Crowe E, Newton RL, Alfonso A, Gordon S, Ryan D (2006). Two-year internet-based randomized controlled trial for weight loss in African-American girls. Obesity (Silver Spring).

[36] Thompson JW, Card-Higginson P (2009). Arkansas' experience: statewide surveillance and parental information on the child obesity epidemic. Pediatrics.

[37] An J, Hayman LL, Park Y, Dusaj TK, Ayres CG (2009). Web-based weight management programs for children and adolescents: a systematic review of randomized controlled trial studies. ANS Adv Nurs Sci.

[38] van Wier MF, Ariëns GA, Dekkers JC, Hendriksen IJ, Smid T, van Mechelen W (2009). Phone and e-mail counselling are effective for weight management in an overweight working population: a randomized controlled trial. BMC Public Health.

[39] (2015). IAP.

