# Supplementary figures and images for: Decreased Body Mass Index in Schoolchildren After Yearlong Information Sessions With Parents Reinforced With Web and Mobile Phone Resources: Community Trial
Source: J Med Internet Res. 2016 Jun 24;18(6):e174. doi: 10.2196/jmir.5584 (PMC4963027; doi:10.2196/jmir.5584)

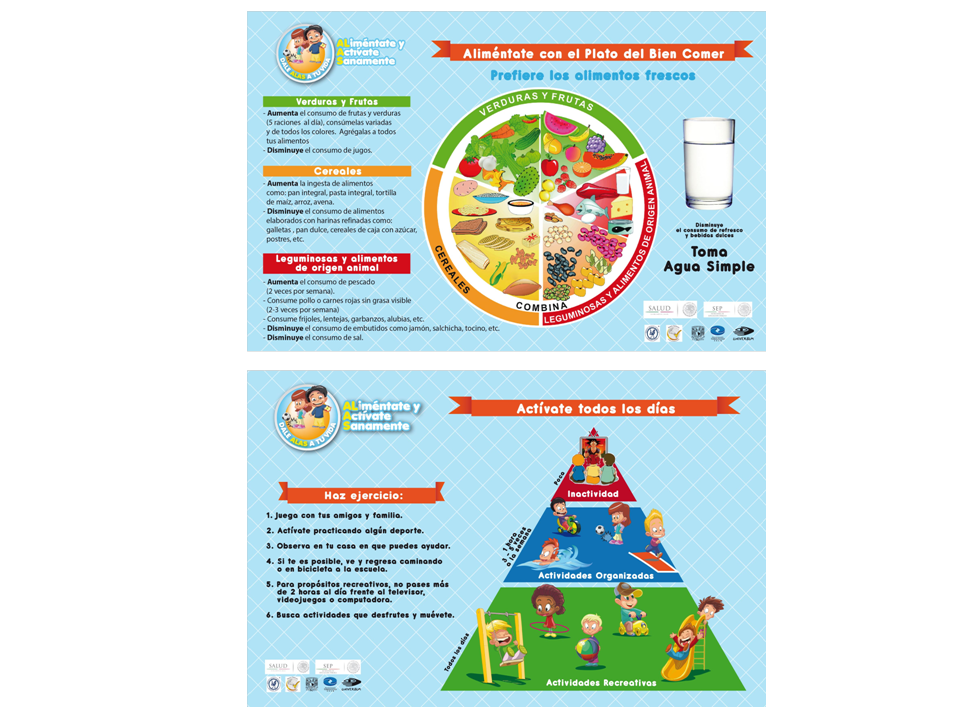

Supplement: Multimedia Appendix 1 [file jmir_v18i6e174_app1.png]

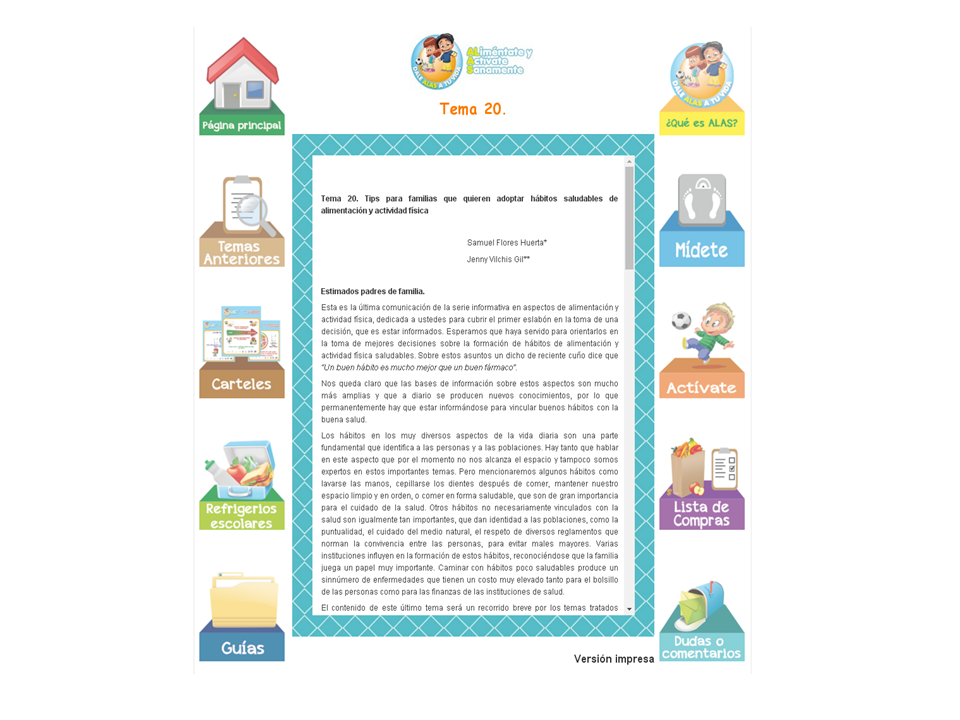

Supplement: Multimedia Appendix 2 [file jmir_v18i6e174_app2.png]

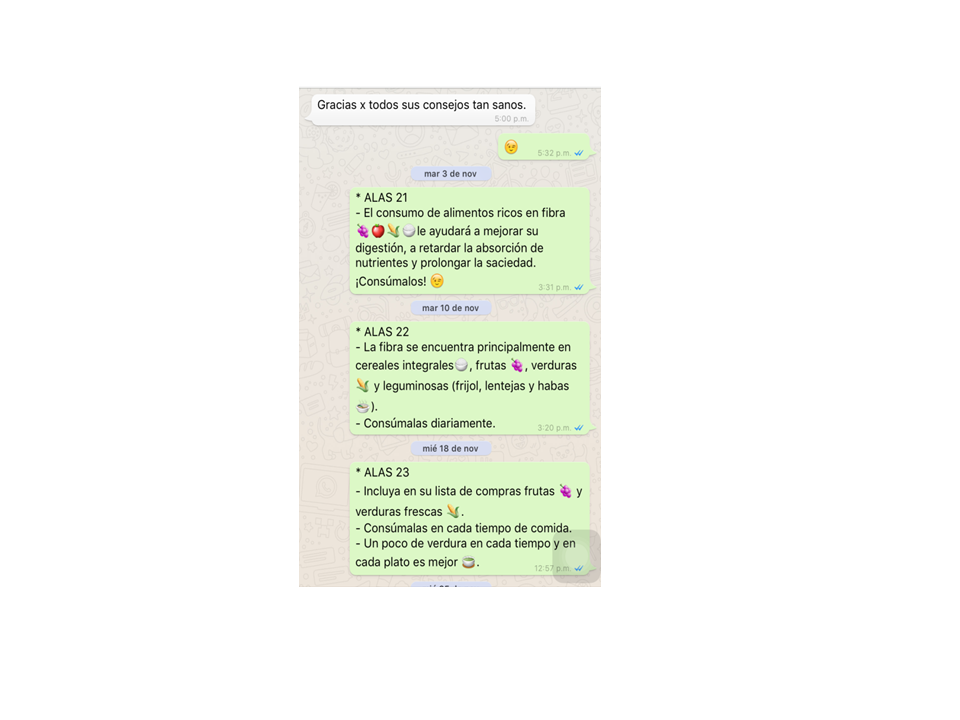

Supplement: Multimedia Appendix 3 [file jmir_v18i6e174_app3.png]
